# The selection by the Asiatic black bear (*Ursus thibetanus*) of spring plant food items according to their nutritional values

**DOI:** 10.3897/zookeys.672.10078

**Published:** 2017-05-04

**Authors:** Shino Furusaka, Chinatsu Kozakai, Yui Nemoto, Yoshihiro Umemura, Tomoko Naganuma, Koji Yamazaki, Shinsuke Koike

**Affiliations:** 1 Tokyo University of Agriculture and Technology, 3-5-8 Saiwai, Fuchu, Tokyo 183-8509, Japan; 2 Kanagawa Prefectural Museum of Natural History, 499 Iryuda, Odawara, Kanagawa 250-0031, Japan; 3 Ibaraki Nature Museum, 700 Osaki, Bando, Ibaraki 306-0622, Japan; 4 Present address: National Agriculture and Food Research Organization, 2-1-18 Kannondai, Tsukuba, Ibaraki, 305-8518, Japan; 5 Present address: Tokyo University of Agriculture, 1-1-1 Sakuragaoka, Setagaya, Tokyo 156-8502, Japan

**Keywords:** Direct observation, feeding ecology, feeding strategy, food habits, nutritional analysis

## Abstract

The present study aimed to investigate the nutritional aspects of the bear diet quantitatively, in order to understand plant food selection in spring. Bears were observed directly from April to July in 2013 and 2014, to visually recognize plant species consumed by bears, and to describe the foraging period in the Ashio-Nikko Mountains, central Japan. Leaves were collected from eight dominant tree species, regardless of whether bears fed on them in spring, and their key nutritional components analyzed: crude protein (CP), neutral detergent fiber (NDF), and total energy. Bears tended to consume fresh leaves of specific species in May, and nutritional analysis revealed that these leaves had higher CP and lower NDF than other non-food leaves. However, CP in consumed leaves gradually decreased, and NDF increased from May to July, when the bears’ food item preference changed from plant materials to ants. Bears may consume tree leaves with high CP and low NDF after hibernation to rebuild muscle mass.

## Introduction

The composition of diets selected by wildlife has long been of interest to range and wildlife biologists. Understanding the reasons underlying food choices is useful when developing and revising habitat management plans (e.g., Aryal et al. 2015a, b, Panthi et al. 2015). Mammals select food types based on relative abundance, seasonal availability, palatability, and nutritional content (e.g., Hanley 1982, Lambert 2010, Aryal et al. 2015a, b). The palatability and nutrient content of plant foods can show rapid temporal variations (Panthi et al. 2015). Therefore, understanding the nutritional basis of dietary selection can help explain feeding phenology.

The feeding habits of bears before and after hibernation (autumn and spring, respectively) are of particular interest (e.g., Noyce and Garshelis 1998, McLellan 2011). Bears show a hyperphagia in autumn for key foods that help build fat reserves for hibernation. In contrast, spring feeding must provide nutrients to rebuild muscle mass lost during hibernation and support lactation in females that are nourishing cubs. American black bears (*Ursus americanus* Pallas, 1780) consume higher protein foods in spring than in summer or fall (Mclellan 2011). This diet facilitates gains in bone and muscle mass in both adult and juvenile bears, with a concurrent loss in fat mass (McLellan 2011, Noyce et al. 1997, Noyce and Garshelis 1998). Because bears are non-cecal monogastric mammals, they are unable to efficiently digest fiber. Thus, the digestibility of plant foods is inversely proportional to their fiber content (Bunnel and Hamilton 1983, Pritchard and Robbins 1990).

Current knowledge suggests that Asiatic black bears (*U. thibetanus* G. Cuvier, 1823) mainly consume green vegetation during spring, such as newly emerged leaves or grasses (Hashimoto and Takatsuki 1997, Hwang et al. 2001). Despite the high volume of green vegetation in the spring diet, vegetative plant remains in feces are difficult to quantify and identify. Most of the previous spring diet studies of Asiatic black bears have relied on fecal analyses, so detailed information on the species consumed is lacking (Hashimoto and Takatsuki 1997). Thus, it also remains unclear what nutritional factors influence the use of food items by Asiatic black bears during spring. Further, bears consume this green vegetation during a limited spring period and then change to other food items quickly (Koike 2010). However, it is unknown why bears select this green vegetation for a short period.

In this study, we aimed to clarify the spring feeding behavior of Asiatic black bears, with a focus on the nutritional factors that contribute to their consumption of spring plant food items. Based on previous studies of American black bears (McLellan 2011, Noyce et al. 1997), we hypothesized that their spring feeding habits are affected by the nutritional components of food items, particularly protein, fiber, and energy. We hypothesized that bears consume plant food items with high protein, high energy, and low fiber to maximize their feeding efficiency during spring. We used direct observations to determine which food items bears consumed and when they consumed them. We then analyzed those food items to determine their nutritional content and elucidated the relationship between nutrient contents and food usage.

## Methods

### Study area

**Figure 1.**
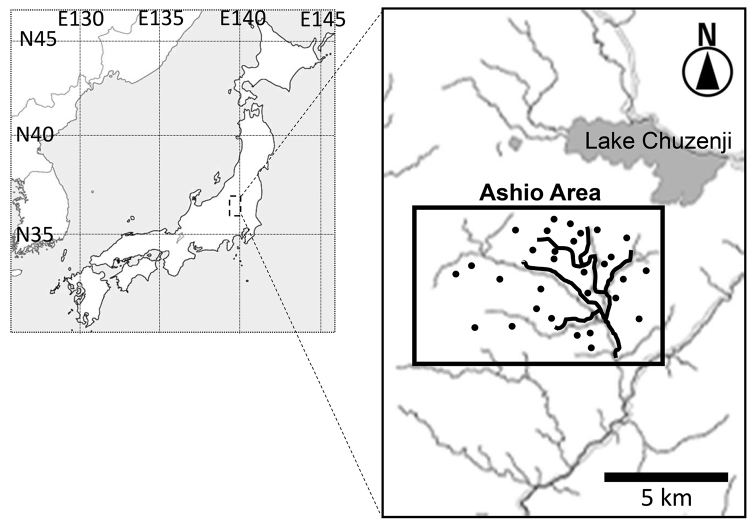
Map of the study area, located in the Ashio-Nikko Mountains range in Tochigi and Gunma Prefectures, central Japan. Black lines were trails to observe bears and black circles were the points of vegetation surveyed in the Ashio area.

This study was conducted in the Ashio area of the Ashio-Nikko Mountains (36°54'–36°80'E, 139°22'–139°49'N; Fig. 1). The main study area was approximately 60 km^2^, with an annual precipitation of 2,236 mm and an annual mean temperature of 7.2°C (Tochigi Prefecture 2011). The natural vegetation of the Ashio-Nikko Mountains up to 1,600 m is deciduous broad-leaved forest composed of *Quercus crispula* Blume, *Acer* spp., and *Fagus crenata* Blume. Mixed forests of *Tsuga* spp. and *Betula* spp. exist above this altitude. Since 1956, the Japanese government has undertaken major tree planting operations in the area, and today, there are scattered stands of *Clethra barbinervis* Sieb. et Zucc., *Alnus firma* Sieb. et Zucc., *Robinia pseudoacacia* Linn., and *Pinus densiflora* Sieb. et Zucc.. However, the landscape remains partly open, with a mosaic of grasslands, rough bare land, planted forest, and natural vegetation.

To clarify the dominant tree species in this area and to select the tree species for analysis, the abundance of tall trees was calculated by a vegetation survey in Ashio study area. Thirty random points were generated in the central Ashio area and set transects (20 × 20 m) in each of the selected points. A handheld GPS receiver (eTrex Legend HCx, Garmin Ltd., Kansas, USA) was used to establish the location where transects were measured. The diameter was measured at chest height of all tall trees and then the total basal area of each tree species was calculated within the transects. The most abundant tall trees in the central Ashio area were *R. pseudoacacia*, *C. barbinervis*, *A. firma*, *Betula ermanii* Cham., and *Q. crispula*.

### Foraging behavior of bears

To identify the food items and foraging period for each food item, bears were directly observed feeding during the daytime from April–July in 2013 and 2014. Most bears showed a diurnal feeding pattern during this season (Kozakai et al. 2013) and, therefore, did little foraging at night. We experienced two types of challenges while using direct observation: it was difficult to locate target animals in this temperate broad-leaved forest and observations had to be made without disturbing the animals or changing their behavior. The steep topography and mosaic grasslands of the Ashio study area enabled us to circumvent these problems; we located bears by visually scanning open grasslands and made our observations from the opposite side of the valley using a video camera with a powerful telescopic lens.

To avoid biases toward certain time frames or individuals, we recorded the feeding behavior for as many different bears as was possible. We were able to identify most of the bears by their physical characteristics including body size, chest markings, and the presence of cubs.

The observation frequency was more than once a week (average ± SD: 1.3 ± 0.5 days/week) depending on weather conditions. We walked along trails in the study area and searched for bears using binoculars (Kenko Skymate 8 × 40 mm) as quietly as possible. When bears were located, their behaviors were recorded using a video camera (Panasonic Lumix GH2) with a telescopic lens. In the laboratory, we reviewed the video recordings to identify each food item by tree shape, tree color, or other characteristics, and to calculate the foraging period for each food item. If we could not identify food items from the video recordings, we returned to the feeding location and directly identified the food items based on the signs of feeding. In addition, particularly during 2014, when observation points were proximate to feeding sites and access to accurate feeding location data (via GPS collars fitted on the bears) was available, we download the GPS location data from GPS collars. This allowed us to visit the feeding locations and assess the food items based on signs of feeding such as bear shelves (broken branches on trees made by bears when eating leaves) or claw marks on tree trunks. We defined “ant-feeding” as occurring when the following conditions were met: a bear turned over a stone, put its nose close to the ground, moved its face up and down, moved its front paws, and these actions had to continue for more than 5 s (Fujiwara et al. 2013). During the survey, to protect from attack by bears, we stayed on mountain slopes opposite from the mountain slopes on which the bears were foraging, generally across the valley, and keeping a minimum distance of 200 m between us and bears.

### Nutritional analysis of food items

From previous studies, we know that Asiatic black bears mainly consume green vegetation, particularly fresh leaves of woody plants, grasses, and herbs in spring (e.g., Hashimoto and Takatsuki 1997). Therefore, we targeted the leaves of woody plants, grasses, and herbs for nutritional analyses during 2013 samplings.

Six food items were identified by direct observation. For the buds or leaves of all tree species (total of four species), the species could be accurately identified by video data based on confirmation (visiting the feeding site and assessing the feeding signs). However, the species of herbs and grasses could not be identified by video data and only used the data of two species that could be confirmed.

The nutritional contents of tree species consumed by bears were compared to those not consumed by bears. We targeted the leaves of woody plants because a previous study indicated that bears in the study area exclusively fed on tree leaves in spring (Koike et al. 2016). We collected leaves from eight tree species (four consumed species and four non-consumed species) with ten individual trees for each species on two separate occasions (early May and late May). The target non-consumed tree species were dominant species (*R. pseudoacacia*, *C. barbinervis*, *A. firma*, and *B. ermanii*), selected based on quantitative data. On the other hand, we observed bears feeding on the leaves of four of these species (*Malus toringo* Sieb., *Q. crispula*, *Salix bakko* Kimura, and *Elaeagnus umbellata* Thund.), and we selected these species as consumed tree species.

Second, to assess the phenologic change in the nutritional value of the three main tree species consumed by bears (*M. toringo*, *S. bakko*, and *Q. crispula*), we collected leaves from ten individual trees of each species once every 2 weeks from leaf flush (early May) to late June (total of four times: early May, late May, early June, and late June). Because feeding on *E. umbellata* leaves was observed less frequently than feeding on other species, *E. umbellata* was excluded from this analysis. Third, whenever we confirmed any other plant species being consumed by bears during our 2013 observations, we collected samples from the foraging site as soon as possible.

After collection, samples (10 g (dry weight) for each tree) were kept in paper envelopes and brought to the Tokyo University of Agriculture and Technology where they were dried at 60°C for 48 h. The dried items were milled, placed in plastic tubes, and stored in desiccators until analysis. For each sample, we determined crude protein (CP, % of dry matter), neutral detergent fiber (NDF, % of dry matter), and total energy content (EN, kcal/gdw) according to the methodology prescribed by the Association of Official Analytical Chemists (2003). We determined the nitrogen content (%) using a CN CODER MT-700 analyzer (Yanaco New Science Inc., Kyoto, Japan) and calculated CP using the following formula: CP = nitrogen × 6.25 (Robbins 1993). We analyzed NDF using an Ankom fiber determination system (Ankom^200^ Fiber Analyzer; Ankom Technology Corp., NY, USA) and analyzed EN using a bomb calorimeter (IKA-Calorimeter C200; IKA-Werke GmbH and Co. KG, Staufen, Germany). All analyses were conducted by the NARO Institute of Livestock and Grassland Science at Tsukuba city, Japan.

After performing nutritional analysis, we used right-angled mixture triangle (RMT; Raubenheimer 2011) analysis to examine the balance of macronutrients and fiber in plant samples. The RMT is a geometric approach used to investigate multidimensional data on the ratios (or balance) of food components in individual foods or food mixtures and is especially relevant to field-based nutritional ecology studies where proportional compositions (as opposed to accurate intake amounts) are the only metric available (Raubenheimer 2011; Raubenheimer et al. 2014). We used a 3-dimensional RMT, where macronutrients were expressed as percentage of total macronutrients (i.e., CP + EN + NDF) on a dry matter basis; CP was shown on the implicit z-axis, the value of which is inversely related with distance from the origin.

### Data analysis

We compared the nutritional content of leaves from four tree species not consumed by bears and from four tree species that bears did consume in early and late May by the Bartlett test and used the Kruskal–Wallis test (KW) to determine the differences in nutritional values between tree species. We also compared the nutritional values of *M. toringo*, *S. bakko*, and *Q. crispula* leaves over time and determined the differences in nutritional values related to phenological change by Bartlett test and KW test. We used a single chi-square test to compare the frequency differences from video recordings of bear foraging times for each food item in each half-month period between 2013 and 2014.

### Results

We recorded bear foraging behavior for 7 h 25 min during 2013 and 8 h 10 min during 2014 (45 individual and 30 individual foraging behavior events during 2013 and 2014, respectively). The number of observed feeding bouts where we could not identify the bear was 68 and 48 during 2013 and 2014, respectively. The minimum number of identifiable individuals was 10 and nine during 2013 and 2014, respectively. We recorded three bears with a GPS collar in 2013 and two in 2014.

During early April, we did not observe any bear feeding behavior, and the leaf flush had not occurred yet. During late April, the leaf flush had still not occurred, and bears mainly ate *Miscanthus sinensis* Andersson. Grass (overwintered culms that had stopped growing in November of the preceding year) [74.7% ± 0.5% (mean ± SD) of video-recorded bear foraging time] and *S. bakko* buds (25.3% ± 0.5%). By early May, the leaf flush had occurred in all tree species except *B. ermanii*. During this period, bears ate newly emerged leaves of *S. bakko* (20.7% ± 0.9%), *M. toringo* (20.3% ± 0.4%), *Q. crispula* (16.6% ± 2.2%), *E. umbellata* (16.1% ± 1.6%), *Eragrostis curvula* (Schrad.) Nees herbs (13.2% ± 2.4%), and unknown grasses (13.1% ± 2.7%). In late May, bears ate the leaves of *S. bakko* (4.7% ± 0.4%) and *Q. crispula* (14.7% ± 0.5%) and the flowers and leaves of *M. toringo* (80.6% ± 0.9%). From June to July, the bears mainly ate ants (96.5% ± 2.1%) and unknown grasses. In early June, bears ate ants (95.0%) and unknown grasses (5.0%) in 2013 and 2014. In late June, bears ate ants (93.9%) and bees (6.1%) in 2013; however, they ate ants (91.5%), unknown grasses (4.5%), and sika deer (*Cervus nippon* Temminck) carcasses (4.0%) in 2014. In early July, bears ate ants (97.2%) and *Cirsium* spp. (2.8%) in 2013; however, they ate ants (94.5%) and unknown grasses (5.5%) in 2014. In late July, bears ate ants (98.0%) and unknown grasses (2.0%) in 2013; however, they ate ants (93.5%), unknown grasses (2.5%), and sika deer carcasses (4.0%) in 2014. There were no large differences in leaf flush timing for any plant species between 2013 and 2014 (all <1 week). There were also no differences between 2013 and 2014 frequencies of video-recorded bear foraging times for each food item during each half-month period, except for late June (*χ*^2^ = 6.586, *P* < 0.05).

During early and late May, EN of food items had no significant effect on consumption; however, bears were significantly more likely to consume food items with a higher CP (KW test: early May: *df* = 6, *P* < 0.05 and late May: *df* = 7, *P* < 0.05) and a lower NDF (KW test: early May: *df* = 6, *P* < 0.05 and late May: *df* = 7, *P* < 0.05). These results indicate that bears use spring plant food items with at least 27% CP and <38% NDF (Table 1).

**Table 1.** Nutritional contents of tree leaves in early and late May.

Species	Early May			Late May		
	EN (kcal/dgw)	NDF (%)	CP (%)	EN (kcal/dgw)	NDF (%)	CP (%)
**Bear consumed plants**						
*Quercus crispula*	**5.2 ± 0.1**	**37.0 ± 5.6**	**31 .8 ± 0.3**	**5.2 ± 0.0**	**26.0 ± 1.4**	**28.4 ± 1.5**
*Salix bakko*	**5.0 ± 0.1**	**20.2 ± 1.0**	**33 .2 ± 4.6**	**5.0 ± 0.2**	**20.3 ± 1.9**	**33.8 ± 0.6**
*Malus toringo*	**5.0 ± 0.1**	**27.9 ± 4.8**	**31.0 ± 2.3**	**4.9 ± 0.8**	**26.7 ± 2 .8**	**27.8 ± 1.6**
*Elaeagnus umbellata*	**5.0 ± 0.0**	**37.4 ± 2.8**	**39.2 ± 1.7**	4.8 ± 0.1	46.2 ± 3.5	38.6 ± 0.8
**No consumed plants**						
*Alnus firma*	5.4 ± 0.1	40.9 ± 2.3	23.1 ± 4.5	5.3 ± 0.1	43.0 ± 2.3	22.9 ± 1.1
*Clethra barbinervis*	4.9 ± 0.4	50.9 ± 19.6	28.3 ± 8.0	4.9 ± 0.3	53.2 ± 1.9	21.1 ± 1.9
*Robinia pseudoacacia*	5.2 ± 0.3	40.5 ± 13.5	27.6 ± 5.6	5.1 ± 0.2	47.6 ± 3.5	25.6 ± 3.5
*Betula ermanii*	–	–	–	5.1 ± 0.3	41.6 ± 0.7	26.3 ± 3.9

Bold type: we can observe that bears consume the food items in that period.

EN, energy content; CP%, crude protein; NDF%, neutral detergent fiber.

**Figure 2.**
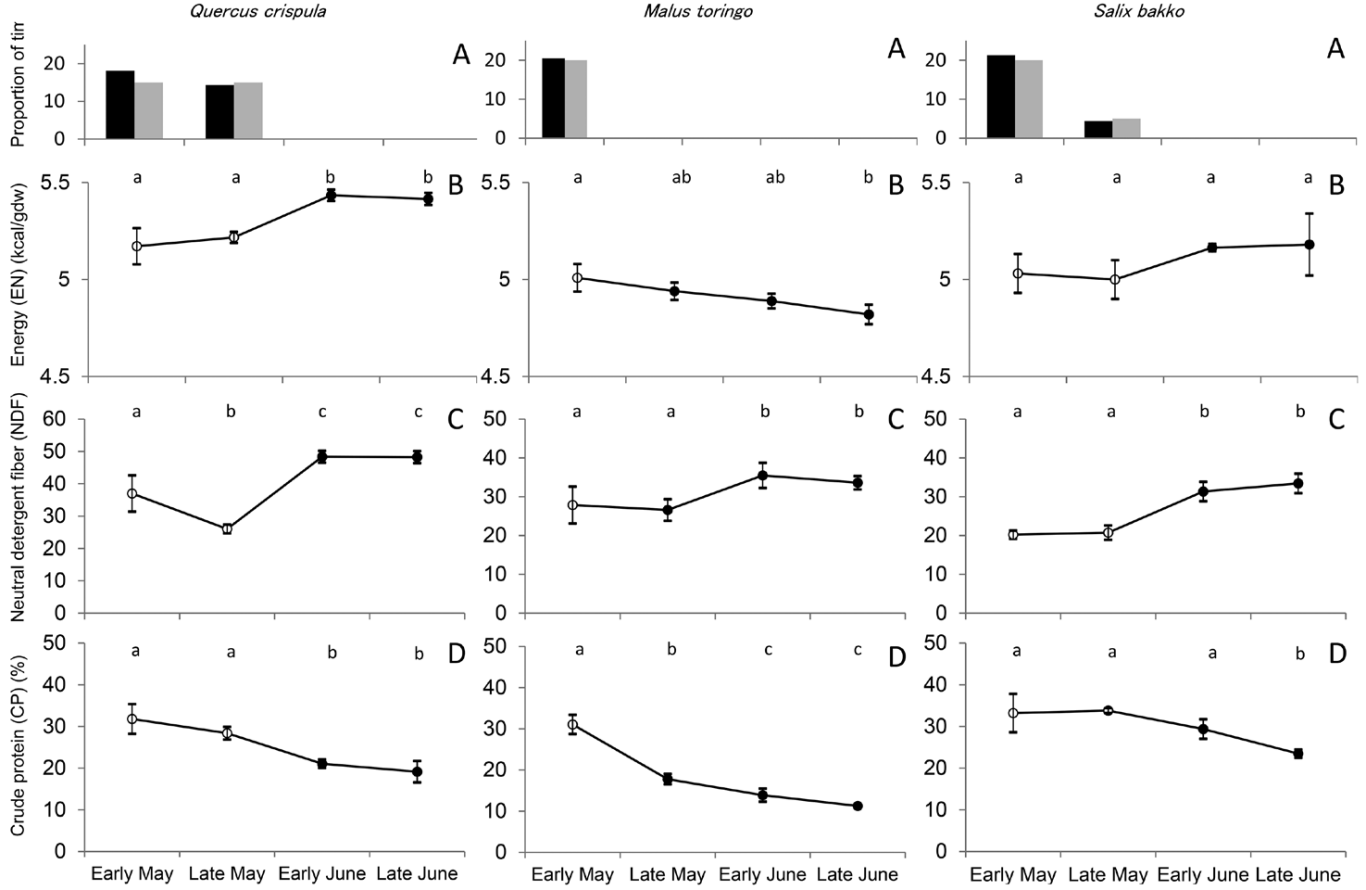
Proportion of observed time for each food item and seasonal changes in the nutritional values of *Quercus crispula* (left) leaves, *Malus toringo* (center) leaves, and *Salix bakko* (right) leaves from early May (leaf flash) to late June 2013. **A** The proportion of time for which bears were observed consuming (2013: black and 2014: gray) **B** total energy **C** neutral detergent fiber, and **D** crude protein. Different lower case letters within each graph indicate significant differences (Kruskal–Wallis test, *P* < 0.05). White circles indicate when bears were observed consuming *Q. crispula* leaves (early May and late May), *M. toringo* leaves (early May), and *S. bakko* (early May and late May).

**Figure 3.**
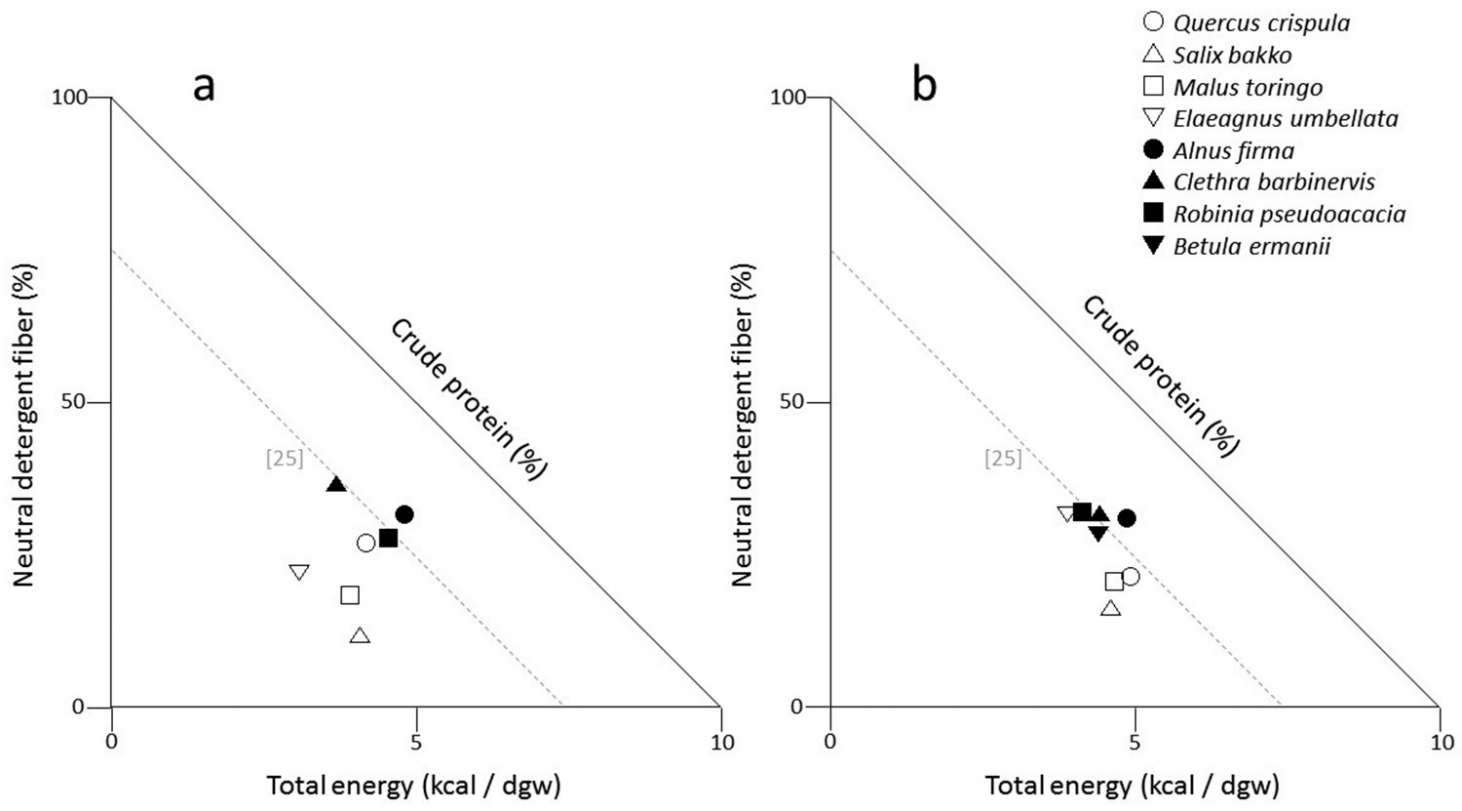
Right-angled mixture triangles (RMT) depicting the macronutrient balance of *Quercus crispula*, *Salix bakko*, *Malus toringo*, *Elaeagnus umbellata*, *Alnus firma*, *Clethra barbinervis*, *Robinia pseudoacacia*, and *Betula ermanii* leaves in early and late May. The RMT on the left **a** is early May, while the RMT on the right **b** is late May. Crude protein is represented on the implicit axis which varies inversely with distance from the origin (the dashed gray line indicates 25% protein content).

In CP, *Q. crispula* leaves decreased from May to late June and was significantly higher in early and late May than in early and late June (KW: *df* = 3, *P* < 0.01; Figs 2, 3). In contrast, NDF increased from May to late June and was significantly lower in early and late May than in early and late June (KW: *df* = 3, *P* < 0.05). EN was significantly lower during early May and early June than during late June and early July (KW: *df* = 3, *P* < 0.01). Bears only consumed the leaves of *M. toringo* in early May (Fig. 1). CP in *M. toringo* leaves significantly decreased for each period from early May to late June (KW: *df* = 3, *P* < 0.01). NDF was significantly lower during early and late May than during early and late June and early July (KW: *df* = 3, *P* < 0.01). EN in *M. toringo* leaves decreased from early May to early July (KW: *df* = 3, *P* < 0.01) and was probably related to the phenology of the leaves. Bears only consumed *S. bakko* leaves in early and late May (Fig. 1). CP in *S. bakko* leaves decreased from May to June and was significantly higher in early and late May and early June than in late June (KW: *df* = 3, *P* < 0.01). NDF was significantly lower during early and late May than during early and late June and early July (KW: *df* = 3, *P* < 0.01). During May and June, EN in *S. bakko* leaves was not significantly different.

Other food items were identified and collected for analysis. In late April, bears consumed dead *M. sinensis* (EN, 4.4 kcal/dgw; NDF, 86.0%; CP, 3.2%) and *S. bakko* buds (EN, 5.0 kcal/dgw; NDF, 24.8%; CP, 45.0%). In late May, bears consumed *M. toringo* flowers (EN, 4.2 kcal/dgw; NDF, 28.3%; CP, 28.4%). The herb *E. curvula* was consumed in early May; however, we were unable to collect samples for analysis.

## Discussion

We found that in spring, Asiatic black bears consumed plant foods with high CP and low NDF. Bears stopped eating the leaves of each of the three tree species when NDF increased above approximately 38%, or CP decreased to less than approximately 27%, which was typical of late spring to early summer. This suggests that CP and NDF are key factors driving the usage of plant food items. EN of foods, per se, was not related to the bears’ choice of foods, suggesting again that a high protein content is the key factor driving bears to eat certain foods, as long as the fiber content is low enough for good digestibility. In contrast, the total energy of a food item may be not an appropriate indicator to explain the diet selectivity of bears.

Adult brown bears (*U. arctos*) accumulate lean mass reserves mostly during spring and early summer (Hilderbrand et al. 2000, McLellan 2011), and rich protein diets during spring enhance this body mass gain (Swenson et al. 2007). Previous studies found that American black bears also gained weight from winter to early summer, suggesting that the protein content of spring food items is of vital importance (Noyce and Galshelis 1998, McDonald and Fuller 2005). In contrast, bears in Japan consume a large quantity of fruits from summer to autumn, many of which contain high levels of available energy, but very low levels of protein and other nutrients (Masaki et al. 2012). Therefore, spring foods containing high levels of protein may be needed to sustain growth throughout the year. However, during late April, bears consumed dead *M. sinensis* that are low in protein and high in fiber. There were exceptions, for example, when limited food items were available and the bears had to consume food items with relatively poor nutritional value. However, the number of these observations was few, and this might be a rare behavior.

Another reason for diet-switching in bears was because more nutritious foods became available. From June to July, the post-season for consuming green vegetation, Asiatic black bears mostly switched to a diet of ants (Fujiwara et al. 2013), which was also seen in our study. A previous study indicated that bears at Ashio could not get sufficient energy from a diet of ants (Yamazaki et al. 2012), thus, the probability of presenting more nutritious foods after June was low in this area, and it is during this period of the year that Asiatic black bears in Japan strip and damage the bark of plantation conifers (Oi and Yamazaki 2006, Kobashikawa and Koike 2016). Although the reasons for this are unclear, research on American black bears suggests that this behavior occurs when their nutritional status is compromised (Flowers 1986). Because the bones and mass of American black bears increase during spring, although there is a loss of fat (Noyce and Garshelis 1998), bears may not be able to replenish lost fat stores on a diet of leaves alone, even if leaves are plentiful during spring. Brown bears in other Asian regions consumed animal materials primarily (Xu et al. 2006, Aryal et al. 2012); therefore, if the bears can get animal materials, they may want to eat more animal materials with higher nutritional quality. In general, when body fat reserves are high, fat is utilized as the main source of energy; however, when fat reserves are low, lean mass is increasingly used as an energy source (Dunn et al. 1998, Caolin 2004, McCue 2010). However, to efficiently maximize the protein in spring plant foods for growth, bears may use their body fat stores remaining after winter to “fuel” their growth, as it takes energy as well as protein to construct new muscle and bone. Future work should focus on how seasonal changes in the availability and nutritional content of food items affect physical conditions such as fat stores.

We recognize the limitations of the present study. First, some food items are easier to observe than others, so focal sampling may be biased toward foods that are easier to observe. Previous food habit research showed that bears ate deer in spring (Koike et al. 2016). We did not observe this behavior, although we may have missed these observations when bears ate under a heavy canopy cover. In contrast, for the first time, most of the vegetative plants were identified and quantified. Second, the results may have been biased toward individual bears, sex, or age classes. However, we observed multiple bears over multiple years; thus, our results may be generally applicable to the bear population in this area. Third, we could not separate the availability of protein and fiber contents in terms of food preferences. Future research should consider other aspects such as changes in biomass (availability) and should also include other food items and other nutritional components.
